# Alcoholic Disease of the Heart

**Published:** 1895-06

**Authors:** Theodore Fisher

**Affiliations:** Registrar, Bristol General Hospital


THE BRISTOL

Medico-Chirurgical Journal.

JUNE, 1895.

### ALCOHOLIC DISEASE OF THE HEART.

BY

Theodore Fisher, M.D., Lond.,

*Registrar, Bristol General Hospital.*

That alcohol influences the heart is one of the best known facts in practical medicine. Where signs of cardiac enfeeblement occur in pneumonia, typhoid fever, or diphtheria, as well as in various phases of chronic heart disease, alcohol is administered. In the majority of such instances alcohol given with discretion doubtless acts beneficially; yet, while a weak heart may gain strength under its influence, one can easily conceive it to be possible that the stimulating powers of alcohol, when it is habitually used in excess, may act injuriously upon a normally acting heart. We not only can conceive such to be possible, but we are well aware that in one phase of chronic alcoholism—viz., delirium tremens—there is danger of sudden cardiac failure, and it is possible that we may have witnessed sudden death in patients suffering from this delirium. Although this liability to heart failure in delirium tremens has long been recognised, it is only within the last few years that alcohol has been suspected of producing a definite cardiac disease. Bollinger of Munich appears to have been the first to draw the attention of the profession to the fact. He noticed that in 1,000 necropsies there were forty-six cases of death with large heart, in which the ordinary causes of hypertrophy, such as valvular disease, arterio-sclerosis, and granular kidney, were absent. He pointed out the excessive beer-drinking of Bavaria generally and of Munich in particular. While in Germany, excluding Bavaria, the amount of beer drunk varied from 54 to 186 litres per head annually, in Munich as many as 432 litres were taken. Bollinger stated that some individuals drank as much as from twenty to thirty litres a day; and in a recent conversation which I had with a German lady in Homburg upon the subject, I was told that the blacksmith of a village in Bavaria, near which she lived, used to take regularly twenty-four quarts of beer a day. Bollinger attributed the cases of large heart for which no recognised cause of hypertrophy could be found to over-indulgence in alcohol, and other writers have since supported him in this conclusion. If one examines carefully-kept *post-mortem* records, such as those of Guy's Hospital, one finds that in past years the pathologist not infrequently adds a note that he can find no cause for a hypertrophied and dilated heart that has led to the death of the patient. On turning up the clinical report of such a case, a history of alcoholic intemperance will probably be found. Brief notes of such cases will be given by me in the forthcoming volume of the Guy's Hospital Reports.

Cases of alcoholic enlargement of the heart, although not generally recognised, are probably far from uncommon. During the past year there have been in the Bristol General Hospital six cases that may reasonably be considered examples of this disease. Three of these ended fatally, and on two autopsies were performed. One of these, under the care of Dr. Markham Skerritt, who has kindly allowed me to mention it, is a fairly typical example. It was a man aged forty-four, admitted October 1st, 1894, who for fourteen years had worked in a brewery, and had taken from eight to nine pints of beer a day. He had suffered from shortness of breath on exertion for two or three years, and while walking had frequently to stop suddenly on account of palpitation of the heart associated with some pain. For three or four months before admission he had left off work. When first seen he was rather stout, with congested face. There was orthopnœa and some œdema of the legs. The heart-impulse was feeble, diffuse, and could not be localised, but was thought to extend outwards beyond the nipple-line. The cardiac dulness was not increased, owing possibly to some emphysema of the lungs. There was no murmur audible, but a well-marked triple sound was heard from the sixth space one-and-a-half inches outside the nipple-line upwards to the third rib and inwards to the left border of the sternum. The pulse was very compressible, of average size, regular (120 to the minute). The liver could not be felt below the ribs. The urine was diminished, the quantity being from twenty-one to twenty-four ounces daily. The patient died suddenly shortly after he had been sitting up talking to a neighbouring patient. At the autopsy a heart weighing seventeen and a half ounces was found. The valves were quite healthy, and the heart-muscle to the naked eye and microscopically showed no abnormal change. The kidneys were also healthy. Here we have a case of death from cardiac failure, and discover a large heart with no valve-disease or kidney-mischief to account for it. Such a case is a more or less typical one of death due to over-indulgence in alcohol.

In some details, of course, cases may vary. Instead of a history of dyspnœa lasting from two to three years as in this case, the patient may have noticed no discomfort until two or three weeks before he is completely incapacitated. Bauer[1] considers that death may occur within a few days from the onset of the cardiac failure. An instance of this occurred in Guy's Hospital. A man who had been a drunkard for twenty-six years suffered from no symptoms of dyspnœa until forty-eight hours before death. Perusal of the Guy's Hospital records, and of a list of twenty-five cases of alcoholic heart-failure published by Dr. Graham Steell,[2] seems, however, to show that a comparatively long history is more common. Although in some instances the history of symptoms pointing to cardiac failure

[1] *Centralbl. f. innere Med.*, 1894, No. 10, quoted in *Practitioner*, vol. liii., 1894, p. 50.

[2] *Med. Chron.*, vol. xviii., 1893, p. 1.

does not date further back than two or three weeks before the patients come under observation, in others it extends into years. Dyspnœa sometimes first manifests itself in sudden nocturnal attacks which, in the absence of disease of the aortic valves, generally point to primary weakness of the heart-muscle, such as is present in this disease.

When habitual dyspnœa is so great that the patient is obliged to take to his bed, the clinical aspect resembles that of other cases of chronic or subacute cardiac failure. The patients being mostly beer drinkers are often rather stout, and possibly cyanosis may be an exceptionally noteworthy feature. In three of the fatal cases that occurred in Guy's Hospital this cyanosis was extreme, and venesection was performed. One of these cases of venesection took place under my direction when I was house physician, and rarely, if ever, have I seen more remarkable cyanosis. Apart, however, from the occasional presence of unusually well-marked cyanosis, the cases do not materially differ from other cases of heart failure. There is the same dyspnœa and orthopnœa, the same œdema of the legs.

So far as physical signs are concerned, since a large heart is present, we shall expect to find evidence of it; and such evidence may exist in the presence of increased dulness and a displaced impulse. Sometimes, however, the impulse cannot be localised, and emphysematous lungs mask the dulness. In such a case the heart-sounds may afford some information. A systolic apex-murmur is common, owing to the mitral orifice sharing in the dilatation of the left ventricle, and a dilated right ventricle may give rise to a tricuspid murmur. A basic systolic murmur, probably also due to dilatation of the right ventricle, may be present. In other cases no murmur may be present, but as in the case mentioned earlier in this paper, a triple sound may be heard. In other cases again neither murmur nor triple sound may be audible, but the heart-sounds may then present some abnormality of tone; and lastly, the sounds may appear quite normal. Dr. Graham Steell points out that the character of the heart-sounds may vary; a murmur may appear and disappear, or may alternate with a triple sound. Some cases in the Guy's

Hospital records reveal the same fact; and at the present time there is a case in the Bristol General Hospital in which there was first a triple sound, then no marked abnormality, after that a tricuspid murmur, and finally again a triple sound. The pulse is generally of low tension, is not uncommonly irregular, and generally of increased rapidity. The liver is commonly enlarged; and Dr. Steell thinks that cases of supposed hypertrophic cirrhosis that have been thought to contract under observation, have probably been congested livers due to alcoholic heart-failure which have diminished in size as the heart has improved. Curiously enough, in spite of the marked alcoholic history in some of the cases of alcoholic heart-failure that occurred in Guy's Hospital, it was the exception to find any cirrhosis of the liver.

It possibly will not be difficult for us to diagnose a hypertrophied and dilated heart; but having settled that point, it may not be easy to decide whether or not it is due to over-indulgence in alcohol. Large hearts associated with mitral valve disease, kidney disease, or affections of the heart-muscle such as fibroid and fatty degeneration, will have to be considered.

I will in a cursory way refer to a few points, and will commence with fatty degeneration. This degeneration, formerly considered not uncommon, can now be said to hardly find a place in medical literature as a distinct disease. Some fatty degeneration is not uncommonly found in the hearts of patients dying from various cardiac diseases, but it rarely, if ever, exists as the only heart-lesion. Occasionally extremely stout people die of heart-failure, and after death their hearts are found covered and infiltrated with fat; but in such cases the general obesity must be considered as a disordered metabolism affecting the whole body, in which the heart has suffered in association with other organs. Cases of heart-failure due to over-indulgence in alcohol no doubt form part of the class of diseases that are frequently diagnosed as fatty degeneration.

While fatty disease of the heart cannot be said to exist, another very definite degeneration—viz., fibroid disease—is not so very uncommon. The frequency with which it leads to sudden death is well known, and we are also familiar with the fact that a common symptom of its presence is severe cardiac pain. Curiously enough, however, it comparatively rarely gives rise to gradual cardiac failure, and cases, therefore, do not often come under observation as clinical cases of heart disease. There was one well-marked case when I was clinical assistant at Guy's Hospital; but perusal of ten years of the *post-mortem* records shows that during that period there were only two other cases of this disease, in which other heart-lesions were absent, admitted to the wards, while no less than ten cases were brought in dead, sudden death having occurred in the streets of London. Should we, however, happen to meet with a case of fibroid disease in which heart-failure is gradual, we shall have the same clinical signs as in a case of alcoholic disease of the heart. Apart from a history of intemperance, we shall in all probability be unable to make a diagnosis.

While cases of fibroid disease are clinically not common, cases of hypertrophy of the heart associated with Bright's disease in which failure has occurred are not infrequently met with. Here it is possible that, in spite of the heart-failure, the presence of a high-tension pulse may render some aid in diagnosis. Unfortunately, however, even this sign may fail us; and instead of a high-tension pulse being present up till the time of death in cases of failing heart associated with chronic kidney-disease, the pulse may be of remarkably low tension. Of recent years it has been customary to lay great stress upon arterio-sclerosis, which so often accompanies kidney disease, as a cause of heart hypertrophy, and possibly the cases in which the pulse remains hard are examples of this affection. Such may be the case; but since arterio-sclerosis rarely exists without some kidney mischief, when a hard pulse is present we may with some reason suspect the presence of renal disease.

There is one class of cases of heart enlargement possibly more closely allied to alcoholic hypertrophy of the heart than any of the foregoing—that is, hypertrophy due to overwork. Here for our diagnosis we must rely upon the history. An arduous occupation will be in favour of the one, intemperance of the other. Three cases that occurred at Guy's Hospital, and one at the Bristol General Hospital, seem to suggest that hard work and heat combined are more potent than hard work under more normal circumstances. The only three fatal cases during ten years at Guy's Hospital were in a stoker, a blacksmith, and a labourer in gas works, and one marked case of hypertrophy of the heart recently in the Bristol General Hospital was also in a stoker.

Possibly in those cases in which a systolic apex-murmur is present, there may be some doubt as to whether or not the case is one of regurgitation due to mitral valve disease. It may here be mentioned that regurgitation from mitral valve disease is not nearly so common as is generally supposed. Mitral stenosis is common enough, but deformity of the flaps of the mitral valve unassociated with contraction of the orifice is rare. For example, in ten years of the Guy's Hospital *post-mortem* records, excluding cases in which disease of the aortic valves was present and adherent pericardium in children, there were sixty-two cases of mitral stenosis, and only five cases of death from mitral regurgitation due to valvular disease. But while mitral dilatation from valve disease is rare, a presystolic murmur is not always heard in cases of mitral stenosis. It was absent in about one-third of the cases. It may be necessary, therefore, to distinguish cases of alcoholic enlargement of the heart in which a mitral systolic murmur is heard from cases of mitral stenosis in which the same murmur only is audible. The following points may be of some value :—Mitral stenosis generally leads to death before the age of thirty-five, whereas alcoholic disease occurs most commonly after that age. Mitral stenosis is most common in women; alcoholic disease, in men. The history of rheumatism or chorea on the one hand, and of over-indulgence in alcohol on the other, may also aid us in arriving at a correct conclusion.

So far as the treatment is concerned, it is possible that the reader may consider this a simple matter. He may think it advisable to treat a case of alcoholic disease of the heart that is sufficiently ill to be confined to bed in the same way as any other case of cardiac disease, with, however, the mental reservation that should the patient return to comparative health, it will be important to impress upon him the necessity of his becoming a total abstainer. Such a line of treatment is adopted by Dr. Graham Steell. He mentions, however, that although he considers that alcohol must be given up at any cost when the heart recovers power, that during the stage of dangerous cardiac failure he thinks that alcohol may be necessary in order to maintain life. Dr. Babcock[1] on the other hand, in a paper upon the treatment of so-called idiopathic hypertrophy of the heart, of which alcoholic disease is a common variety, takes somewhat different views. In the first place he thinks rest in bed should, if possible, be avoided. He considers that the circulation is hampered rather than aided by physical quiet, since muscular action and comparatively deep respiratory movements no longer materially aid in propelling the blood onward. Dr. Babcock goes farther, and considers digitalis, the drug so useful in most heart affections, to be harmful, and states that it "has no place in the treatment of this affection." He thinks nitrate of strychnia valuable, and inclines to the stimulants recommended by Fraentzel, such as musk, castoreum, and valerian. He also believes the treatment by baths and exercises to be especially useful in these cases, and has treated cases with marked benefit.

That the treatment by baths and exercises is useful in these cases I cannot doubt. The cases that Dr. Theodore Schott kindly allowed me to see and examine at Nauheim last month seemed to be chiefly cases of so-called idiopathic hypertrophy of the heart, in some of which there was a suspicion of alcoholic intemperance. Through kind permission of Dr. Schott, I am able to briefly refer to one or two.

(1) A schoolmaster, aged forty-five, for five years had suffered from cardiac pain and dyspnœa, and for several months before going to Nauheim had been compelled to give up teaching. He was then unable to walk for five minutes. He was treated last year for about five weeks at Nauheim, and had returned when I saw him for a short course. He could, he said, walk about six miles. Examination of the heart showed that the impulse was slightly displaced outwards, but nothing abnormal was heard with the exception of a faint tricuspid systolic murmur. There was reason to suspect alcoholic intemperance as the cause of the heart-trouble.

[1] *J. Am. M. Ass.*, vol. xxiii., 1894, p. 940.

(2) A manufacturer, a stout, finely-made man, aged about fifty, was first sent five years before to Dr. Schott by Professor Gerhardt, of Berlin. At that time he could not walk a few yards without pain and dyspnœa. When I saw him he could walk eight miles without difficulty. Since first going to Nauheim he had paid two short visits there every year. Examination of the heart did not reveal any obvious hypertrophy, but at the point of impulse there was a localised systolic murmur. Dr. Schott suspected over-indulgence in alcohol in this case, and said that lately the patient had noticed its harmful effects upon his heart.

(3) A tall, well-made man, a railway inspector, aged forty-eight, about three years before had commenced to suffer from palpitation, with dyspnœa on exertion, and occasionally at night. In September, 1893, he was hurriedly walking to a station when he fell down in a fainting fit, which lasted some hours. He was taken to the hospital at Tübingen, and admitted under the care of Professor Liebermeister, who said that the heart was dilated and discovered a murmur. The following year he visited Nauheim, where he was greatly benefited. I could detect nothing abnormal in the size of the heart, or in its sounds. In this case also Dr. Schott had reason to suspect alcohol.

(4) A wine merchant, aged about forty-five, had suffered from dyspnœa on exertion for about two years, and last year was attacked with nocturnal dyspnœa. In September he came for two or three weeks to Nauheim, at the end of which time he had lost his nocturnal dyspnœa and could walk up a hill about 300 feet high. When I saw him shortly after his return in the spring some irregularity of the pulse was present, there was obvious enlargement of the heart and a systolic apex-murmur. Here the occupation of the patient suggests the possibility of over-indulgence in alcohol.

Difficult though it may be to explain the effect of these baths and of the associated exercises, there can be no doubt that patients suffering from some forms of heart disease derive great benefit. For example, one patient told me that before going to Nauheim he could only walk a few yards, but that after his first visit he had walked fourteen miles. Before visiting the baths it seemed probable that I should find the majority of cases that materially improve would be cases of cardiac hypertrophy unassociated with valve-disease, and my experience confirmed such a view. A common cause of such hypertrophy appears to be over-indulgence in alcohol; and, as already mentioned, it was found that Dr. Schott had reasons for suspecting this indulgence in some of the cases that he kindly showed me.

Here in England, however, we cannot treat all cases by baths and exercises, and those cases that are sufficiently ill to require confinement to bed would perhaps be hardly in a condition for such treatment. Dr. Graham Steell, in adopting the routine treatment for heart disease in his cases, lost only four out of twenty-five; yet the fact that soon after complete confinement to bed sudden death may occur in patients who do not appear to be in any immediate danger, as took place in the case mentioned at the commencement of this paper, seems to show that there are some grounds for Dr. Babcock's surmise that absolute rest may be harmful. Careful observation of the effect of rest or gentle exercise, of digitalis or strychnine, of alcohol or it may be the expensive musk, will lead us to decide as to the best treatment to pursue in any individual case.

